# Sorangicin A Is Active against *Chlamydia* in Cell Culture, Explanted Fallopian Tubes, and Topical In Vivo Treatment

**DOI:** 10.3390/antibiotics12050795

**Published:** 2023-04-22

**Authors:** Simon Graspeuntner, Katharina Koethke, Celeste Scholz, Lea Semmler, Mariia Lupatsii, Laura Kirchhoff, Jennifer Herrmann, Katharina Rox, Kathrin Wittstein, Nadja Käding, Lars C. Hanker, Marc Stadler, Mark Brönstrup, Rolf Müller, Kensuke Shima, Jan Rupp

**Affiliations:** 1 Department of Infectious Diseases and Microbiology, University of Luebeck, 23538 Luebeck, Germany; simon.graspeuntner@uksh.de (S.G.);; 2 German Center for Infection Research (DZIF), Partner Site Hamburg-Lübeck-Borstel-Riems, 23538 Lübeck, Germany; 3 Helmholtz Centre for Infection Research (HZI), Helmholtz Institute for Pharmaceutical Research Saarland (HIPS), and Department of Pharmacy, Saarland University, 66123 Saarbrücken, Germany; 4 German Center for Infection Research (DZIF), Partner Site Hannover-Braunschweig, 38124 Braunschweig, Germany; 5 Department of Chemical Biology, Helmholtz Centre for Infection Research, 38124 Braunschweig, Germany; 6 Department of Microbial Drugs, Helmholtz Centre for Infection Research, 38124 Braunschweig, Germany; 7 Department of Obstetrics and Gynecology, University Hospital of Schleswig Holstein, 23538 Luebeck, Germany

**Keywords:** sorangicin A, antibiotics, *Chlamydia trachomatis*, *Chlamydia muridarum*, mouse, novel therapeutics, microbiota, vagina

## Abstract

Current treatment of *Chlamydia trachomatis* using doxycycline and azithromycin introduces detrimental side effects on the host’s microbiota. As a potential alternative treatment, the myxobacterial natural product sorangicin A (SorA) blocks the bacterial RNA polymerase. In this study we analyzed the effectiveness of SorA against *C. trachomatis* in cell culture, and explanted fallopian tubes and systemic and local treatment in mice, providing also pharmacokinetic data on SorA. Potential side effects of SorA on the vaginal and gut microbiome were assessed in mice and against human-derived *Lactobacillus* species. SorA showed minimal inhibitory concentrations of 80 ng/mL (normoxia) to 120 ng/mL (hypoxia) against *C. trachomatis* in vitro and was eradicating *C. trachomatis* at a concentration of 1 µg/mL from fallopian tubes. In vivo, SorA reduced chlamydial shedding by more than 100-fold within the first days of infection by topical application corresponding with vaginal detection of SorA only upon topical treatment, but not after systemic application. SorA changed gut microbial composition during intraperitoneal application only and did neither alter the vaginal microbiota in mice nor affect growth of human-derived lactobacilli. Additional dose escalations and/or pharmaceutical modifications will be needed to optimize application of SorA and to reach sufficient anti-chlamydial activity in vivo.

## 1. Introduction

**Figure 1:**
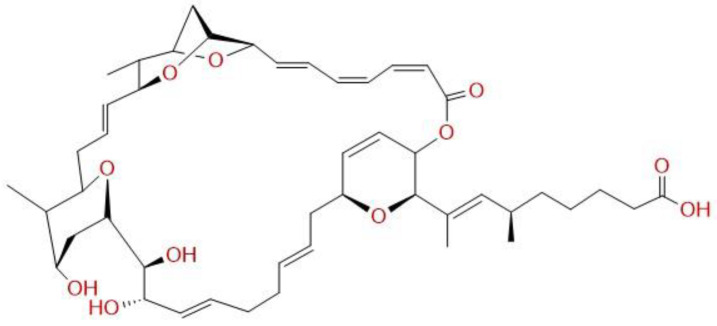
Chemical structure of Sorangicin A.

More than 100 million are the global estimated incident cases of genital *C. trachomatis* infections [1], which makes the intracellular pathogen the most common sexually transmitted infection (STI) amongst various other bacterial STIs [2]. While generally infecting both men and women, women carry the major burden of the illness. Chlamydial infections, especially when recurrent, cause upper genital sequelae, such as pelvic inflammatory diseases (PID), ectopic pregnancy, and infertility [3]; thus, antibiotic treatment of the infection is necessary. Current first-line treatment options are oral application of 100 mg doxycycline twice a day for one week or one single dose of 1.5 g azithromycin. However, azithromycin has certain limitations that in part account for an estimated treatment failure rate of ~11% [4,5] and both antibiotics are detrimental to resident *Lactobacillus* species [6]. Consequently, new antibiotic agents and strategies should be investigated. While established treatment strategies focus on systemic drug application, no topical therapies against vaginal chlamydial infections are so far available. However, antibacterial topical therapeutics are well established in special fields, such as ophthalmology and dermatology [7,8], providing benefits of easy application, lower substance concentration, and avoidance of systemic side effects. By investigating novel anti-chlamydial substances, e.g., corallopyronin A, we could show high effectiveness of this substance in in vitro and ex vivo models [9]; however, its systemic effectiveness in vivo could not be proven [10]. Exhibiting the same target as corallopyronin A for its antibacterial mechanism, we now analyzed the anti-chlamydial effects of the antibiotic sorangicin A (SorA). The compound was originally isolated from *Sorangium cellulosum*, a member of the bacterial order myxococcales, a group of bacteria known for the production of antimicrobial substances [11]. SorA (Figure 1) acts by inhibiting the bacterial transcription via blocking the beta-subunit of the bacterial RNAP specifically, preventing RNA translocation [12] (a general description of anti-bacterial mechanisms targeting RNAP can be found in a recent paper by Wenholz et al. [13]). Therefore, the compound shows selective, broad spectrum antibacterial activities in vitro and is particularly active against Gram-positive pathogens [12]. Intracellular bacteria, such as *Mycobacterium tuberculosis,* was also shown to be susceptible to SorA treatment [14]. Yet, effects of SorA application on *C. trachomatis* remained unclear and its in vivo capacities are to be studied.

Thus, we investigated SorA’s effectiveness against chlamydial infections in cell culture, in human fallopian tissue and in a mouse model. Further, we were interested in the potential harmful side effects of the new drug on the commensal microbes and analyzed its impact on the vaginal and gut microbiome in our mouse model and *Lactobacilllus* species from the human vagina.

## 2. Materials and Methods

### 2.1. Bacterial Strains and Human Cells

*C. trachomatis* serovar D (ATCC VR-885) and *C. muridarum* NiggII (ATCC VR-123) were purchased from ATCC and propagated in HeLa (ATCC) or HEp-2 (ATCC) cells in vitro. Strains were regularly checked for strain-specific inclusion morphology and growth kinetics, and an amplicon sequencing approach is available at the lab to determine strain specificity. Human fallopian tubes were infected ex vivo with *C. trachomatis* serovar D. *C. muridarum* NiggII was used for all in vivo experiments for the genital infection of the mice.

### 2.2. Chemicals

Doxycycline was purchased from Sigma-Aldrich (St. Louis, MO, USA). SorA (produced by the Helmholtz Zentrum für Infektiologie (Braunschweig, Germany)) was diluted in dimethyl sulfoxide (DMSO). For in vitro and ex vivo experiments, a stock solution (*c* = 5775 µg/mL) was prepared and stored in aliquots at −20 °C. Work solutions were prepared directly before experimental runs. Experiments of topical application were performed by using a mixure of the SorA-DMSO solution (10%), PBS (60%), and macrogolglycerol ricinoleate (30%). For intraperitoneal experiments, the SorA-DMSO solution (10%) was mixed with PBS (90%).

### 2.3. Determination of the Minimal Inhibitory Concentration (MIC) for Chlamydiae

A total of 0.5 × 10^5^ HeLa cells was grown in each well of a 24-well plate (Greiner bio-one, Frickenhausen, Germany) in RMPI 1640 with 5% FCS, non-essential amino acids and 2 mM glutamine without antibiotics (Sigma-Aldrich Corporation, St. Louis, MO, USA) for 24 h at 37 °C and 5% CO_2_. Afterwards, cells were infected with *C. trachomatis* serovar D, followed by centrifugation (800 × g, 1 h, 35 °C). Infection was titrated to yield an infection rate of approximately 60% in DMSO-treated controls. Infected cells were then incubated with or without indicated SorA concentrations under normoxic (20% O_2_) or hypoxic (2% O_2_) conditions at 37 °C, and negative controls were treated with the solvent DMSO. MICs were determined by visualization of the growth of chlamydial inclusions after 30 h of incubation based on n = 3 experiments. Chlamydial inclusions were visualized by immunofluorescence staining with a mouse anti-chlamydial lipopolysaccharide (LPS) antibody (kindly provided by Prof. Helmut Brade, Borstel, Germany) and a polyclonal rabbit FITC-labeled anti-mouse IgG antibody (Dako Denmark A/S, Glostrup, Denmark).

### 2.4. Testing Recoverable Chlamydiae

For recovery assays, a total of 3 × 10^5^ cells/mL HEp-2 cells was used in 24-well plates and incubated for 24 h at 37 °C with 5% CO_2_. Infected HeLa cells of the primary infection were separately treated with indicated concentrations of SorA. Afterwards, cells from primary infection were washed with medium to remove the remaining SorA. The cells were scratched from the surface and homogenized in tubes using glass beads on a vortexing device. Recoverable chlamydiae were determined, as described previously [15], by serial dilutions of the homogenized primary infection on recovery plates. Recovery plates were incubated for 30 h and chlamydial inclusions were visualized as described above.

### 2.5. Assessing Growth Pattern Changes of Species from the Genus Lactobacillus under Sorangicin A Application

To analyze whether the growth pattern of lactobacilli would be altered upon SorA application, six patient-derived vaginal isolates from our STI culturomics facility [16] were selected: *L. gasseri*, *L. fermentum*, *L. jensenii*, *L. mucosae*, *L. reuteri*, and *L. rhamnosus*. Culture purity was confirmed via MALDI-TOF (matrix assisted laser desorption/ionization time of flight) (Bruker Corporation, Billercia, USA) and a pre-culture inoculated in 10 mL brain heart infusion media (Thermo Fischer, Waltham, MA, USA) under anaerobic conditions (Whitely H35 HEPA, Don Whitley Scientific, Bingley, UK). After 24 h of growth, the optical density (600 nm) of precultures was measured (Nanophotometer P330, Implen GmbH, München, Germany). Based on the turbidity, equivalent volumes were added to 10 mL brain heart infusion media. Next, 200 μL of media with inoculum were transferred to the 96 multi-well plates (96 flat bottom, Greiner, Kremsmünster, Austria) and SorA solution was added to selected wells (end concentration 100 ng/mL). Brain heart infusion media with and without SorA were used as a negative control. Bacterial growth patterns were measured (Epoch2 multi-plate reader, BioTek, Winooski, VT, USA) every 30 min for 72 h (37 °C, 282 cpm rotation, 600 nm). Growth rate was calculated while comparing the first measured point with growth at 20 h using following formula: (log (growth at timepoint 2) − log (growth at timepoint 1)) × 2.303 (timepoint 2 − timepoint 1). Growth rate values were used after deblanking.

### 2.6. Efficacy of Sorangicin A against C. trachomatis Serovar D in the Human Fallopian Tube Ex Vivo Model

Preparation of human fallopian tubes was performed as described previously [17]. Briefly, the tissue of human fallopian tubes was collected from individuals undergoing hysterectomy by a trained physician. Fallopian tubes were made available to the study if no macroscopically visible signs of inflammation were apparent based on the judgement of the physician. A separate fallopian tube for each replicate was dissected in a Petri dish containing RPMI1640 (Gibco/Invitrogen, Schwerte, Germany) with 5% FCS (Gibco/Invitrogen) removing connective tissue and tissue destroyed by the surgery. After dissection, human fallopian tubes were opened with a scalpel. The tissue was infected with 5 × 10^8^ IFUs *C. trachomatis* serovar D and treated with or without (1 or 2 µg/mL) SorA before 48 h of incubation. Afterwards, tissue pieces were washed with medium to remove remaining SorA. The tissue was homogenized using glass beads in a homogenizer and recovery assays of *C. trachomatis* were determined.

### 2.7. Application of Sorangicin A in an In Vivo Mouse Model of Chlamydia Muridarum Infection

Eight-week-old female C57BL/6JRj mice (Janvier Labs, Le Genest-Saint-Isle, France) were synchronized to the same stage of the estrous cycle by subcutaneous injection of 2.5 mg medroxyprogesterone acetate (Depo-Clinovir^®^, Pfizer, New York, NY, USA) per mouse. After 7 days, each mouse was vaginally infected with 10^6^ IFUs *C. muridarum* (as conducted previously [10]) or mock-infected with sucrose phosphate buffer (SPG). SorA was applied every 12 h either from day 1 to 7 after infection or from day 4 to 11 after infection. Doxycycline (50 mg/kg body weight) and the respective vehicle were used as control groups for intraperitoneal treatment. For intravaginal treatment, SorA or doxycycline diluted in DMSO (10%), PBS (60%), and macrogolglycerol ricinoleate (30%) were used. A SorA concentration of 5 or 10 mg/kg body weight was intravaginally applied in a volume of 21.4 µL. After each application, the mouse was restrained upside down for one minute. For intraperitoneal application, 35 mg/kg SorA was used in 150 µL solution of DMSO (10%) and PBS (90%). Vaginal swabs were collected directly before infection at days during and after chlamydial infection, and recovery assays of *C. muridarum* were performed on HEp-2 cells to determine the bacterial burden.

### 2.8. Microbiome Analysis

Stool droppings and vaginal swabs were taken on a regular basis during the above-mentioned mouse experiments. We used swabs that were opened together with the actually used swabs but remained unused to control for DNA-contamination. For microbiome analysis of theses samples, DNA isolation was performed using the DNeasy PowerSoil Kit (Qiagen, Düsseldorf, Germany). Each round of isolation was complemented with an isolation control containing all reagents to account for potential introduction of DNA-contamination at this stage as well. From the isolated DNA, PCR was performed amplifying the V3/V4 region of the bacterial 16S rRNA gene. Specialized primers [18] were designed as we described previously [19]. All primers targeted the region 319F or 806R of the bacterial 16S rRNA gene. Primers included a heterogeneity spacer and an index sequence. Every single specimen was assigned to a unique combination of indices to barcode each sample during PCR, making it suitable for sequencing. Continuing, gel electrophoresis was utilized to determine DNA concentration and size of amplicons on an aliquot of each sample. Following, all barcoded samples were put together from the original PCR into one complete library containing DNA concentration of 50 ng per sample, run through gel electrophoresis, and bands were picked with Gene Catcher tips on a pipette. These gel pieces were further eluted with the MinElute Gel Extraction Kit (Qiagen, Düsseldorf, Germany) following the manufacturer’s protocol. To quantify the concentration of the eluted samples, we used the NEBNext^®^ Library Quantification Kit for Illumina^®^ (New England Biolabs, Ipswich, Massachusetts, United States) following the manufacturer’s instructions. Finally, all sequencing procedures were performed using the sequencing-by-synthesis technique on an Illumina MiSeq (Illumina, San Diego, CA, USA) desktop sequencer.

Raw sequencing reads were processed using mothur [20] version 1.44.1 via the following pipeline: sequences with homopolymers under 12 and sizes shorter than 500 bp were aligned against SILVA reference data base [21], not aligned sequences were removed from further analysis. Chimeric sequences (VSEARCH [22]) were removed and remaining sequences were taxonomically assigned using the Ribosomal Data Base [23] or (if species level classification was desired) using EzBioCloud [24]. Statistical analysis and graphical visualization were assembled via R (version 4.0.1) using package vegan [25]. Alpha diversity measurements were assessed using Shannon’s diversity index and calculating the number of detected taxa in each of the sample types. Indicator species analysis was used to reveal taxa being associated with SorA treatment.

### 2.9. Measurements of SorA Levels

To analyze SorA concentration in vaginal cells, the same type of swabs as for recoveries was taken over the course of the experiment from uninfected control animals with topical SorA treatment. Vaginal cell swabs for tissue levels were taken on day 2 and 9. Directly after swab collection, the swab was put into a 1.5 mL reaction tube and shock frozen in liquid nitrogen before storage in −80 °C. For tissue analysis of the genital tract, three nine-week-old mice were topically treated with SorA for seven days as previously described; however, they were not infected before treatment. One hour after the last application of SorA, all mice were sacrificed, followed by immediate dissection of the genital tracts. The genital tracts were separated in uteri, adnexa, and vagina samples and stored in 1.5 mL reaction tubes and put into liquid nitrogen.

Vaginal swabs were extracted by adding 500 µL of a mixture of 20% DMSO and 80% of a L-ascorbic acid solution (20/80 (*v*/*v*)) and extraction for 2 h at 1000 rpm on a vortex mixer in the dark. Samples were homogenized using 3 mL of 0.9% isotonic sodium chloride solution with an ULTRA-TURRAX^®^. All samples were analyzed via HPLC-MS/MS using an Agilent 1290 Infinity II HPLC system coupled to an AB Sciex QTrap 6500+ mass spectrometer. First, a calibration curve was prepared by spiking different concentrations of SorA into a mixture of 20% DMSO and 80% L-ascorbic acid solution (20/80 (*v*/*v*)) (as matrix for vaginal swabs), or homogenized organs. Caffeine was used as an internal standard. We used 100 µL of each vaginal swab and 20–50 µL of homogenized tissue. Next, 200 µL MeOH was added and samples were concentrated for 5 h in the dark using an Eppendorf concentrator. A mixture of 200 µL ACN + 195 µL of a 20% L-ascorbic acid solution + 5 µL caffeine (1 µg/mL in ACN) was used as an extraction agent, added to the samples, shortly vortexed, extracted for additional 5 min at 800× *g* on a vortex mixer, and then centrifuged for 5 min at 16,000× *g* at 4 °C. The supernatant was transferred to HPLC glass vials for analysis. Mass spectrometric conditions were as follows: scan type: MRM, positive mode; Q1 and Q3 masses for caffeine and SorA can be found in Supplementary Table S1; and peak areas of each sample and of the corresponding internal standard were analyzed using MultiQuant 3.0 software (AB Sciex, Toronto, Canada). Peak areas of the respective sample of SorA were normalized to the internal standard peak area. Peaks of PK samples were quantified using the calibration curve. The accuracy of the calibration curve was determined using QCs independently prepared on different days. Experiments for SorA plasma level measurements were performed by Saretius (Reading, UK).

### 2.10. Statistics

Readouts for primary infection and recovery data were analyzed by one-way ANOVA, followed by post hoc tests corrected for multiple testing using Holm–Sidak or using the Tuckey’s test as provided in GraphPad Prism. For longitudinal recovery data and microbial alpha diversity from mouse experiments, we used two-way ANOVA followed by pairwise post hoc tests with correction for multiple testing. SorA levels were tested with a similar strategy, but with one-way instead of two-way ANOVA.

## 3. Results

### 3.1. Sorangicin A Is Active against C. trachomatis in Cell Culture and the Fallopian Tube Model

**Figure 2:**
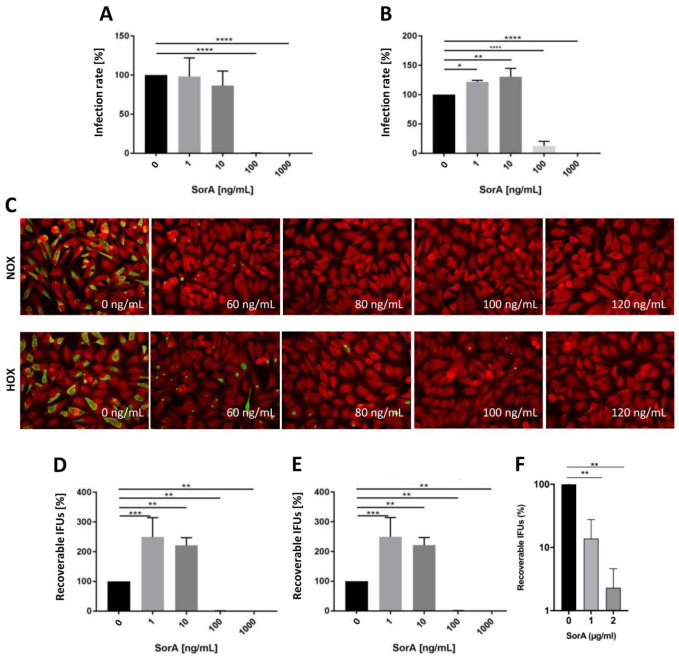
SorA displays high efficacy against *C. trachomatis* in cell culture and an ex vivo fallopian tube model. A primary infection screen of *C. trachomatis* on HeLa cells shows significant reduction in detectable chlamydial inclusions at 100 ng/mL for both normoxic (**A**) and hypoxic (**B**) environments (n = 3, ANOVA multiple comparison test with Holm–Sidak correction and significances from * *p* < 0.05, ** *p* < 0.01, and **** *p* < 0.0001). MIC was assessed as shown by representative pictures of a SorA-treated infection model among normoxic and hypoxic conditions (**C**). Among normoxic conditions, no chlamydial inclusions are detected at a SorA concentration from 80 ng/mL, as among hypoxic conditions from 120 ng/mL (green = chlamydial inclusions, red = HeLa cells, n = 3). Recovery of *C. trachomatis* showed a significant reduction in the infectious progeny from the infection at a SorA concentration of 100 ng/mL and higher for both normoxic (**D**) and hypoxic (**E**) environments (n = 3, ANOVA multiple comparison test with Holm–Sidak correction and significances from ** *p* < 0.01, and *** *p* < 0.001). When using a human fallopian tube model (**F**), significant reduction in chlamydial recovery was achieved at the tested condition of 1 µg/mL and 2 µg/mL (n = 3, one-way ANOVA with multiple comparison and Tukey post hoc correction).

To determine if SorA has anti-chlamydial activity, we compared the ability of *C. trachomatis* to form inclusions in the presence of SorA in HeLa cells. Using a logarithmic range of concentration from 1 ng/mL up to 1 µg/mL, we identified significant reduction in *C. trachomatis* inclusions at 100 ng/mL in both normoxic (Figure 2A) and hypoxic (Figure 2B) conditions. We subsequently determined that the MIC was in the range of 60 ng/mL to 120 ng/mL SorA, with a median MIC of 80 ng/mL SorA under normoxia and of 120 ng/mL SorA under hypoxic conditions.

While inclusion formation and MIC are important measures for the efficacy against intracellular *Chlamydia*, we further analyzed the effects of SorA on *C. trachomatis* progeny in HeLa cells. Similar to the effects of SorA on inclusion formation, 100 ng/mL SorA significantly reduced the yield of infectious *C. trachomatis* from the primary infection under both normoxic and hypoxic condition. For sub-inhibitory SorA concentrations, recoverable inclusion forming units (IFUs) were higher compared with untreated controls in normoxic and hypoxic environments (Figure 2D,E). We further investigated the efficacy of SorA in a previously established ex vivo tissue model under normoxic conditions. Treatment with 1 µg/mL SorA significantly reduced *C. trachomatis* progeny in the fallopian tube model (Figure 2F).

### 3.2. Efficacy of Topical and Systemic Sorangicin A Application on Chlamydial Shedding Differs in a Mouse Infection Model

**Figure 3:**
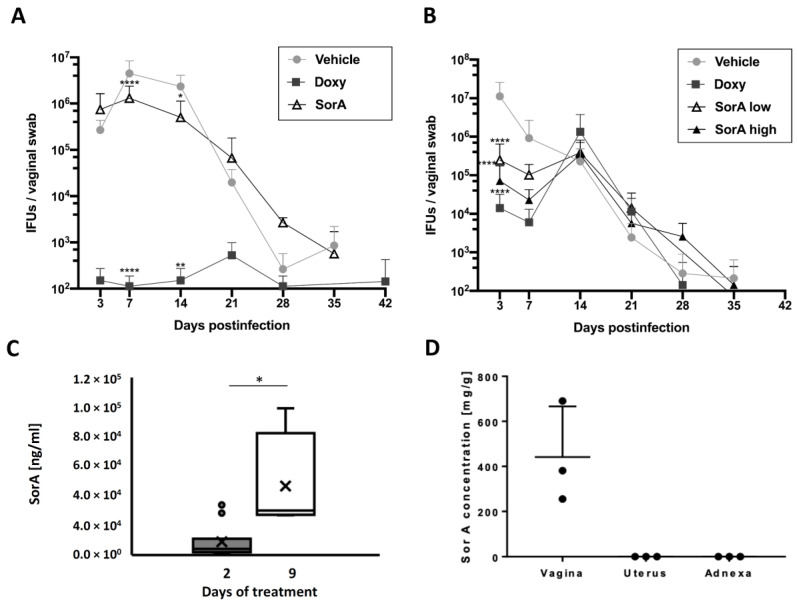
Topical SorA treatment decreases the initial *C. muridarum* burden in a mouse model. Chlamydial shedding from the vagina was only slightly reduced by systemic treatment with 35 mg/kg body weight SorA (**A**), while the anti-chlamydial effect was much more pronounced under topical treatment in a dose-dependent manner (**B**) when using 5 (SorA low) or 10 (SorA high) mg/kg body weight (n = 4, two-way ANOVA multiple comparisons and significances vs vehicle control from * *p* < 0.05, ** *p* < 0.01, and **** *p* < 0.0001). Doxycycline (Doxy) was applied as a positive control using 50 mg/kg body weight for panels (**A**,**B**). SorA was measured from vaginal cell swabs (**C**) by high-performance liquid chromatography after topical application, indicating prolonged application leading to an accumulation of SorA in the vagina (n = 11 at day 2, n = 4 at day 9, Wilcoxon rank sum test, * *p* < 0.05). However, tissue levels of SorA in n = 3 animals following topical treatment (**D**) reveal SorA to be restricted to the site of application (data given as individual data points including mean + standard deviation).

While in vitro cell culture experiments and the ex vivo fallopian tube model demonstrated the efficacy SorA displays against *C. trachomatis*, we next sought to check its efficacy in an in vivo mouse model. While systemic application of 35 mg/kg SorA elicited only a minor reduction in *C. muridarum* recovery from vaginal swabs during the course of infection (Figure 3A), topical application of SorA elicited a much more significant reduction (Figure 3B). Notably, increasing doses of the topically applied SorA treatment from 5 mg/kg to 10 mg/kg further decreased *C. muridarum* recovery (Figure 3B). Mimicking the physiological situation during an infection in humans, we also delayed the antibiotic treatment until days 4 to 11 after infection and observed a similar but less pronounced efficacy compared to simultaneously treated mice (Supplementary Figure S1).

### 3.3. Accumulation of Sorangicin A Is Detected at the Site of Topical Treatment Only

We next analyzed SorA levels in the vagina in uninfected mice. Analysis of SorA levels from vaginal swabs on day 2 of topical SorA treatment showed its presence in the vagina (mean: 8.9 µg/mL, standard deviation: 11.4 µg/mL) and accumulation of the antibiotic on day 9 when treatment time was increased (mean: 46.5 µg/mL, standard deviation: 35.2 µg/mL) (Figure 3C). SorA could be detected only at the direct site of application (ranging from 84.9 up to 230.1 µg/g in vaginal tissue) and not at upper parts of the urogenital tract (Figure 3D). Of note, intraperitoneally injected SorA was cleared from the serum within 7 h of administration (Supplementary Figure S2), explaining at least in part the missing anti-chlamydial effectivity in this model.

### 3.4. Impact of Sorangicin A on the Gut and Vaginal Microbiota

**Figure 4:**
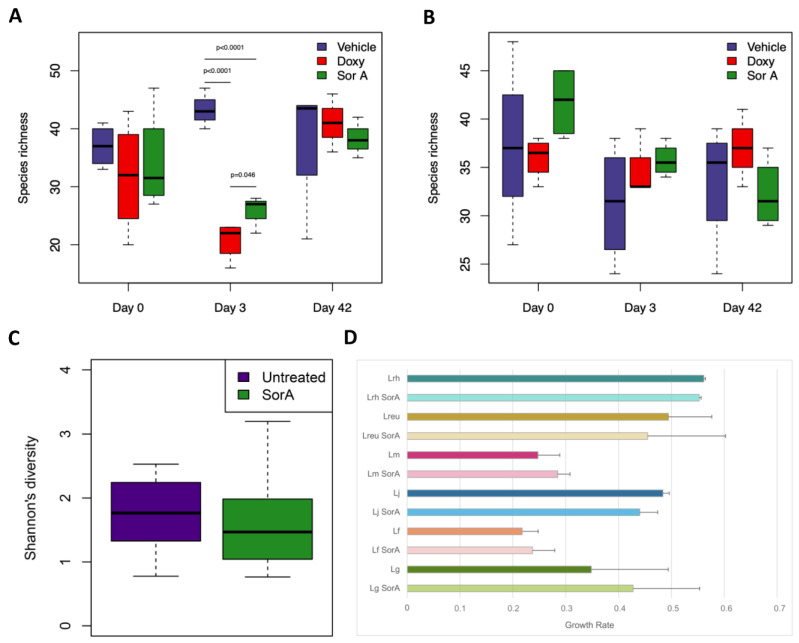
Topical administration of SorA preserves gut microbial composition and does not harm vaginal microbiota. Gut microbial species richness was analyzed before infection (day 0), during antibiotic treatment (day 3), and at termination of the experiments (day 42), showing that antibiotic treatment induces a transient decrease in number of taxa at day 3 only following intraperitoneal treatment (**A**) with 35 mg/kg body weight SorA and 50 mg/kg body weight Doxycycline (Doxy). In contrast, topical treatment (**B**) with 5 mg/kg body weight SorA and 50 mg/kg body weight Doxycycline (Doxy) did not impact gut microbiota (n = 8, two-way ANOVA followed by pairwise post hoc tests with correction for multiple testing). Microbiome analysis from vaginal swabs of uninfected mice (**C**) shows no change in microbial diversity during topical SorA application (n = 8, paired Student’s *t*-test). Single bacterial isolates from human vaginal swabs show no growth difference (**D**) when subjected to growth under standard conditions versus 100 ng/mL SorA (resembling MIC values for *C. trachomatis*) in brain heart infusion media (n = 3, unpaired Student’s *t*-test adjusted for the number of tested bacteria by Holm correction). Growth rate was calculated by comparing optical density (OD) of first-time measured time point with OD at 20 h (log (growth at timepoint 2) − log (growth at timepoint 1)) × 2.303 (timepoint 2 − timepoint 1). Lrh: *Lactobacillus rhamnosus*; Lreu: *L. reuteri*; Lm: *L. mucosae*; Lj: *L. jensenii*; Lf: *L. fermentum*; and Lg: *L. gasseri*.

A thriving question in modern development of new antibiotic treatment strategies is potential side effects on the residual microbiota. We compared the impact of SorA treatment after intraperitoneal and topical administration on the gut microbiota. Intraperitoneal injection of SorA significantly reduced the number of observed taxa (Figure 4A), whereas topical application of SorA in the vagina did not impact the richness of the gut microbiota (Figure 4B). Of note, topical treatment did not significantly alter the microbial diversity within the vagina when tested in uninfected animals (Figure 4C). We further performed an indicator species analysis comparing vaginal microbiota of the mice before and under treatment with SorA. Interestingly, while no other bacterial taxa were found to be significantly associated with SorA treatment or the control group, SorA treatment was associated with detection of *Lactobacillus taiwanensis* in the indicator species analysis (*p*-value 0.02), with respective reads being only found in the SorA-treated group. In addition, we analyzed the effectivity of SorA at a concentration of 100 ng/mL, resembling the mean MIC for *C. trachomatis*, on the growth of six different *Lactobacillus* sp. derived from human vaginal samples (*L. gasseri*, *L. fermentum*, *L. jensenii*, *L. mucosae*, *L. reuteri*, and *L. rhamnosus)*. We could show that the growth of the tested lactobacilli is not impaired by SorA treatment (Figure 4D).

## 4. Discussion

Although genital tract infections with *C. trachomatis* can be efficiently treated and cured with currently available antibiotics, the burden of infections has continuously increased [1], followed in some cases by severe clinical entities, such as salpingitis or PID [3]. Additionally, diagnostic restraints in detecting asymptomatic infections, antibiotic treatment failures, and recurrences of infections under azithromycin are common [5]. Even though emergence of antimicrobial resistance in is not a problem for *C. trachomatis* so far, first choice antibiotics against *C. trachomatis* were shown to negatively affect vaginal microbiota composition, and in particular, *Lactobacillus* species [6]. This illustrates the need for novel antibiotic substances and treatment strategies against *C. trachomatis* to both help circumventing treatment failures and provide an alternative not being harmful to the resident vaginal bacteria. The macrolide-polyether-antibiotic SorA inhibits the bacterial transcription via blocking the DNA-dependent RNA polymerase specifically. The substance was proven active against Gram-positive bacteria and mycobacteria, and in higher concentrations also against Gram-negative bacteria [12]. It was also effective in inhibiting growth of intracellular bacteria, such as *Mycobacterium tuberculosis* [14]. Within the group of selective RNAP inhibitors from cultures of myxobaceria, Corallopyronin A, which has a different binding site than SorA, was found to be highly efficacious against chlamydial infections [9,10], yet lacks effective in vivo application [10]. Interestingly, sorangicins are more potent than corallopyronins in vitro. While they do have the same binding site as rifampin [26], they are effective even against rifampin-resistant *M. tuberculosis*, which is important to consider in a broader perspective when developing new treatment strategies. We therefore tested SorA against *C. trachomatis* in cell culture in a human fallopian tube model as well as in an established *Chlamydia* mouse infection model, including vaginal and gut microbiota analysis for potential side effects, as well as pharmacological determination of SorA tissue levels.

We could show earlier that efficacy testing of antibiotics differs with respect to the availability of oxygen. As cervical and vaginal oxygen concentrations range from 0.5 to 5.5% [27,28] and can be affected by pathophysiological conditions (e.g., bacteria-induced inflammation [29]), SorA was tested among hypoxic (2%) and normoxic (20%) conditions in cell culture experiments. We determined that MICs against *C. trachomatis* serovar D were 80 ng/mL under normoxic and 120 ng/mL under hypoxic conditions, with consecutive recovery assays displaying high anti-chlamydial potency. The observed SorA concentrations are comparable to previous studies with Corallopyronin A, representing similar anti-chlamydial properties [10]. When compared to first-line anti-chlamydial drugs, such as azithromycin and doxycycline, comparable values of 125 ng/mL for azithromycin and 30 to 63 ng/mL for doxycycline are found [15,30,31]. Oxygen depletion resulted in increased MICs of SorA, suggesting a reduced intracellular activity of the antibiotic among low oxygen conditions, as it was also shown for first line treatment before [15,32]. While the increased MIC-value under hypoxic condition is to be considered in further planning when developing SorA as a new treatment option, the observed MIC-value should not qualify as an obstacle in the efficacy of the substance in vivo. Interestingly, sub-inhibitory concentrations of 1 and 10 ng/mL SorA slightly increased infection rates and recovery of infectious particles, which is a so far not-well-understood phenomenon also described for cefotaxime treatment against *Salmonella typhimurium* infections [33]. While the appearance of such effects in an organism remains unclear, this points towards the importance of reaching sufficient concentration in the tissue where the infection resides.

Reflecting more the physiological situation of female infections, SorA was additionally tested in an ex vivo human fallopian tube model. This model is frequently used to transfer findings from cell culture or mice to a more complex model of genital tract chlamydial infections in humans [17,34,35,36]. In accordance with the in vitro findings, SorA at concentrations of 1 to 2 µg/mL significantly reduced chlamydial growth and progeny in human fallopian tubes, pointing at the need to closely monitor the required dose to be reached in the infected tissue.

To analyze the systemic effect of SorA on chlamydial shedding in the urogenital tract, we performed mice infection experiments with *C. muridarum*, as an established rodent model in *Chlamydia* research [37,38,39]. SorA was either applied systemically (i.p.) or topically via intravaginal administration. Intraperitoneal SorA application against *C. muridarum* showed only a minor reduction in chlamydial growth. Measurements of SorA plasma levels in mice revealed a relatively short disposition of the drug within the murine blood stream, presumably not reaching respective drug concentrations above the MICs in the genital tract. Along that line, dose response and drug stability experiments are needed to evaluate the potential of SorA for systemic application against *Chlamydia* [40]. However, SorA may also just not be secreted to the female genital tract after systemic antibiotic application, as other antibiotic investigations showed that plasma concentrations would not always be indicative of the antibiotic efficacy in the target tissue [41]. In principle, intraperitoneally applied SorA is systemically transported and active, which could be proven by analyzing the stool microbiota of these animals. Treated animals showed a significant decrease in observed bacterial taxa following intraperitoneal SorA administration, indicating a potential harmful, untargeted side effect on the gut microbiome. It was pointed out how adverse collateral damage on the gut microbiome by well-established antibiotics can be [42]; therefore, we consider acknowledging strategies to circumvent such damage already in the developmental process of new antibiotic substances a key point to consider.

In contrast to systemic application of SorA, topically applied SorA in mice revealed an elicited reduction in the chlamydial load within the early stage of infection (Supplementary Table S2 compares the main achievements of the models used within this work). Topical application of e.g., antifungals, is widely used in the vagina or to treat bacterial vaginosis [43], but targeted antibiotic treatments are better explored and established in the field of ophthalmology and dermatology. Topical treatments are easily applied and can be used with lower drug concentrations compared with systemic administration. In addition, it can help to avoid systemic side effects, such as vomiting, the first-past effect, a feared consequence of oral antibiotic treatment, and the development of gut microbiome dysbiosis [44], which we could also observe for SorA. In this line, topical SorA application does not have negative side effects, neither on the gut nor on the vaginal microbiota. Importantly, SorA at MIC concentration for *C. trachomatis* does also not inhibit the growth of various tested vaginally isolated *Lactobacillus* species, making SorA potentially superior to first-choice antibiotics regarding preservation of protective microbes in the vagina.

## 5. Conclusions

Although topical SorA application significantly reduced chlamydial load in the murine vagina, the observed effect was less pronounced than in doxycycline-treated animals and it is not to be expected that the detected reduction in SorA-treated animals would have a strong impact on ascending chlamydial infection and pathology. A broad range of models and test conditions is needed to extract strength and weaknesses of a novel substance and to deduce at an early stage whether additional efforts towards clinical applicability are promising. For SorA, further analysis should be focused on specific factors that apparently impair substance stability in the vaginal tract after intravenous and intraperitoneal administration. Given that myxobacteria do present to produce a large number of antibacterial substances, screening closely related analogs of SorA may prove useful. Modification of antibacterial substances [45] or drug conjugation to biological carriers [46] may also improve efficacy of SorA within the urogenital tract. It may further be important to consider that host proteins are playing a role in sensitizing to antibiotics [47]. Given the fact that *Chlamydia* infection was shown to reduce the host’s translation machinery [48] there may be further mechanisms reducing the effectivity of SorA treatment in vivo to be encountered. Addressing those points will be a prerequisite to test the efficacy of SorA against chlamydial pathology in ascending infections in the mouse model in comparison to the currently applied treatment regiments.

## Data Availability

The sequencing data used within this study are being made available at the European Nucleotide Archive with accession number PRJEB55639.

## Supplementary Materials

The following supporting information can be downloaded at: https://www.mdpi.com/article/10.3390/antibiotics12050795/s1, Figure S1. Topical SorA treatment only slightly decreases the *C. muridarum* burden during the course of an established infection in a mouse model. Figure S2. Plasma levels after intravenous and intraperitoneal SorA application. Table S1. Q1 and Q3 masses for caffeine and SorA. Table S2: Contribution of the different models to developing sorangicin A as a new therapeutic option for the treatment of chlamydial infections.
