# Supplementary material for: Sorangicin A Is Active against Chlamydia in Cell Culture, Explanted Fallopian Tubes, and Topical In Vivo Treatment
Source: Antibiotics (Basel). 2023 Apr 22;12(5):795. doi: 10.3390/antibiotics12050795 (PMC10215493; doi:10.3390/antibiotics12050795)
Supplement: Supplementary file 1 [file antibiotics-12-00795-s001.zip › antibiotics-2350125-supplementary.pdf]

antibiotics

MDPI

Article

Sorangicin A Is Active Against *Chlamydia* in Cell Culture and Vaginal Topical Antibiotic Treatment in Mice

Simon Graspeuntner [1,2,†], Katharina Koethke [1,†], Celeste Scholz [1], Lea Semmler [1], Mariia Lupatsii [1], Laura Kirchhoff [1], Jennifer Herrmann [3,4], Katharina Rox [4,5], Kathrin Wittstein [4,6], Nadja Käding [1,2], Lars C. Hanker [7], Marc Stadler [4,6], Mark Brönstrup [4,5], Rolf Müller [3,4], Kensuke Shima [1] and Jan Rupp [1,2,*]

[1] Department of Infectious Diseases and Microbiology, University of Luebeck, 23538 Luebeck, Germany; simon.graspeuntner@uksh.de (S.G.)
[2] German Center for Infection Research (DZIF), Partner Site Hamburg-Lübeck-Borstel-Riems, 23538 Lübeck, Germany
[3] Helmholtz Centre for Infection Research (HZI), Helmholtz Institute for Pharmaceutical Research Saarland (HIPS), and Department of Pharmacy, Saarland University, 66123 Saarbrücken, Germany
[4] German Center for Infection Research (DZIF), Partner Site Hannover-Braunschweig, 38124 Braunschweig, Germany
[5] Department of Chemical Biology, Helmholtz Centre for Infection Research, 38124 Braunschweig, Germany
[6] Department of Microbial Drugs, Helmholtz Centre for Infection Research, 38124 Braunschweig, Germany
[7] Department of Obstetrics and Gynecology, University Hospital of Schleswig Holstein, 23538 Luebeck, Germany
* Correspondence: jan.rupp@uksh.de
† These authors contributed equally to this work.

Supplementary material content:

Supplementary Figure S1, Supplementary Figure S2, Supplementary Table S1, Supplementary Table S2

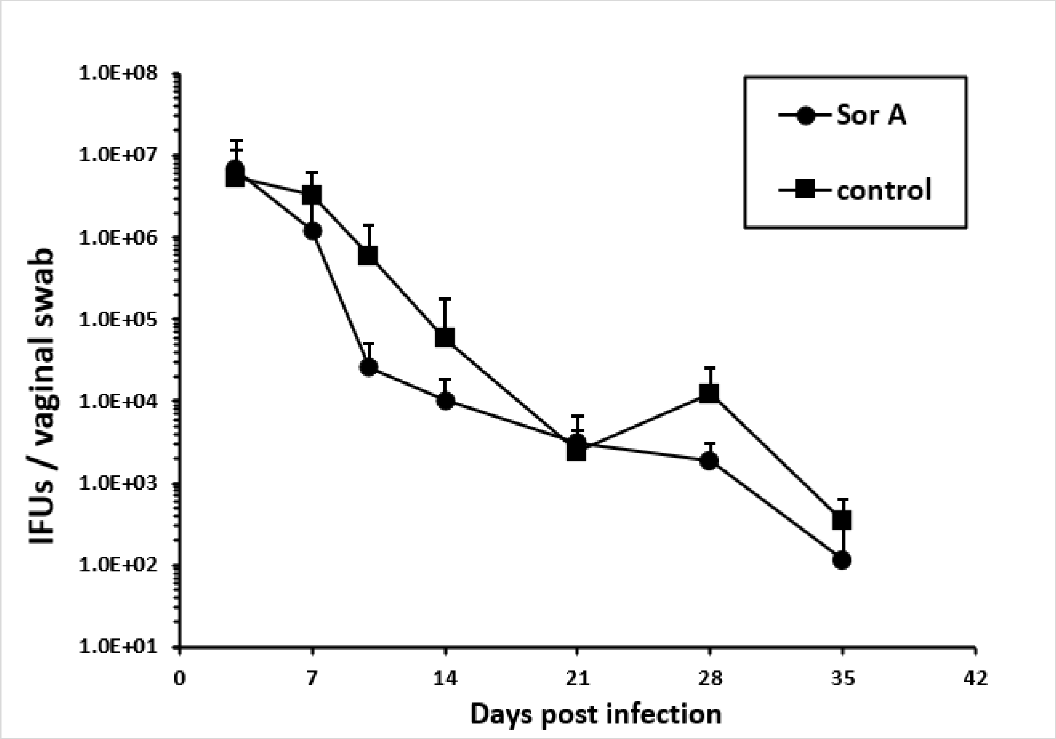

Supplementary Figure S1. Topical SorA treatment only slightly decreases the *C. muridarum* burden during the course of an established infection in a mouse model. Chlamydial shedding from the vagina established a peak at day 3 post infection but was reduced when SorA was applied topically from day 4 – 11. Application of SorA was performed twice daily using 5 mg/kg body weight was intravaginally applied in a volume of 21,4 µl. (n=5 two-way ANOVA multiple comparisons and significances vs vehicle control, *p<0.05). Red line indicating start of treatment.

A **B**

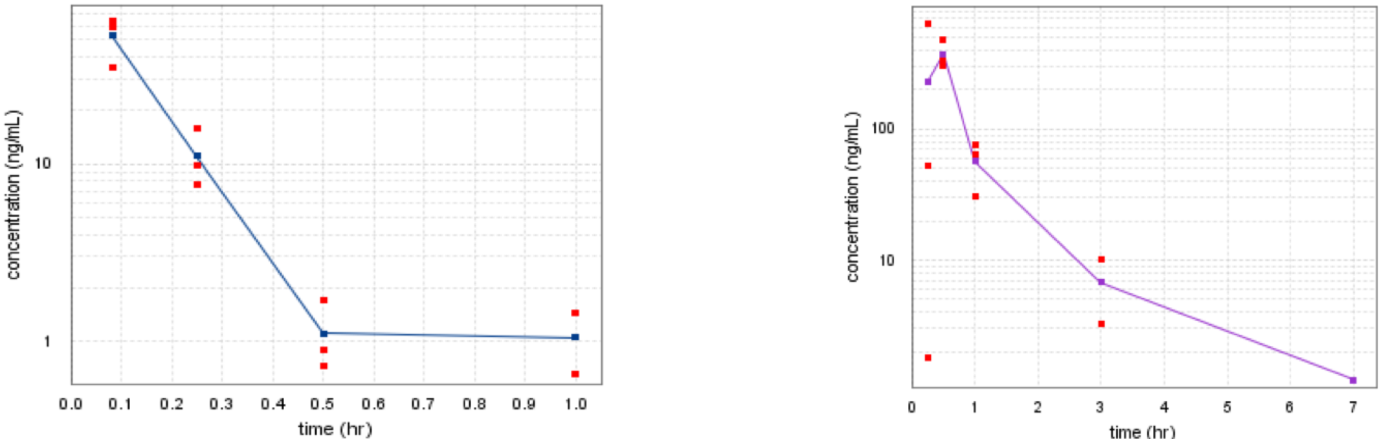

Supplementary Figure S2. Plasma levels after intravenous and intraperitoneal SorA application. In (A) 9.51 mg/kg Sor A was applied intravenously. One hour after intravenous application of Sor A, the average plasma concentration of the substance successively decreased from circa 14 ng/ml to around 1 ng/ml after half an hour and then to not detectable concentrations after another 30 minutes. (B) 46.3 mg/kg Sor A was injected intraperitoneally. After an initial increase of the plasma concentration, it decreased from a maximum plasma concentration of 120 ng/ml to below detection limit by 7 hours after application. hr: hours (n=3), red dots: individual measurements, blue dots/lines: mean values.

Supplementary Table S1. Q1 and Q3 masses for caffeine and SorA

ID	Q1 Mass [Da]	Q3 Mass [Da]	time [msec]	DP [volts]	CE [volts]	CXP [volts]
**SorA**	824.441	789.3	50	51	19	20
**SorA**	824.441	771.3	50	51	21	20
**SorA**	824.441	753.3	50	51	25	20
**SorA**	824.441	807.3	50	51	13	20
**caffeine**	195.024	138.0	50	60	25	14
**caffeine**	195.024	11.0	50	60	31	18

Supplementary Table S2: Contribution of the different models to developing sorangicin A as a new therapeutic option for the treatment of chlamydial infections.

Item	Cell culture (human)	Ex vivo fallopian tube model (human)	Systemic application (mouse)	Topical application (mouse)
**Eradication of C. trachomatis**	Achieved	Achieved	Not achieved	Partially achieved
**Protection of vaginal microbiota**	NA	NA	Achieved	Achieved
**Protection of gut microbiota**	NA	NA	Not achieved	Achieved
